# Studying the Process of Phosphogypsum Recycling into a Calcium Sulphide-Based Luminophor

**DOI:** 10.3390/nano14110904

**Published:** 2024-05-22

**Authors:** Oleg A. Medennikov, Marina A. Egorova, Nina P. Shabelskaya, Asatullo Rajabov, Sergey I. Sulima, Elena V. Sulima, Zlatislava D. Khliyan, Daniil I. Monastyrskiy

**Affiliations:** 1 Department of Ecology and Industrial Safety, Faculty of Technology, Platov South-Russian State Polytechnic University (NPI), Novocherkassk 346428, Russia; monomors@yandex.ru (O.A.M.); m.egorova@npi-tu.ru (M.A.E.); nina_shabelskaya@mail.ru (N.P.S.); rajabov.asadullo@mail.ru (A.R.); zlata.tkachenko.98@mail.ru (Z.D.K.); danya.monastyrskij.95@mail.ru (D.I.M.); 2 Department of Chemical Technologies, Faculty of Technology, Platov South-Russian State Polytechnic University (NPI), Novocherkassk 346428, Russia; elena-sulima66@mail.ru

**Keywords:** phosphogypsum, recycling, reduction, citric acid, ultraviolet luminophore

## Abstract

Currently, one of the most important problems of environmental protection is the deep and complex processing of mineral raw materials. This problem is especially relevant when processing substandard ores and production waste, one of which is phosphogypsum. This study examines the process of CaSO_4_/CaS composite material formation during the reduction of phosphogypsum with citric acid. The composite structure formation mechanism is proposed. The resulting materials are characterized using various methods, including X-ray diffraction (XRD), transmission electron microscopy, the Scherrer method, thermogravimetric analysis (TGA), and FT-IR spectroscopy. The reduced sample emits orange radiation in the range of 500–750 nm with a quantum yield of 0.17. Experimental results showed that the sample decomposition process in the solid state consisted of two components with a predominant contribution from the long-lived component (~46 ns). The optimal conditions for producing luminescent materials by reducing phosphogypsum with citric acid were determined: a heat treatment temperature of 1073 K, a holding time of 60 min, and a reducing agent mole fraction of 37%. It was found that an increase in temperature with a simultaneous decrease in heat treatment time, as well as a decrease in temperature with a simultaneous increase in heat treatment time, led to a decrease in the luminescent properties of the synthesized material compared to optimal values. The results can be used to develop technology for recycling large-tonnage waste from the chemical industry into luminescent materials.

## 1. Introduction

The current level of production throughout the world is directly related to the global use of natural resources and the accumulation of large-tonnage technogenic waste [1,2,3]. This is a reality that must be considered; even an essential technically advanced industrial complex, if its impact on nature extends beyond environmentally acceptable limits or becomes destructive, may become undesirable for society, if not today, then in the nearest future.

Currently, one of the most important problems of environmental protection is the deep and complex processing of mineral raw materials. Several studies are devoted to ore materials’ complex processing issues and increasing the environmental safety of ore-related production [4,5,6]. This problem is especially relevant when processing substandard ores and production waste, including phosphogypsum.

Phosphogypsum is a product formed during the decomposition of raw phosphate materials with sulfuric acid using an extraction method. As a rule, extraction of phosphorus from raw materials is carried out with mineral acids (phosphoric, sulfuric, or, less often, nitric), which produce large quantities of waste. In recent years, many attempts have been made to recycle phosphogypsum. Among them, several areas can be noted. The authors in [7] proposed a technology for the complex processing of phosphogypsum into a chemical ameliorant. The use of phosphogypsum has been shown to improve the growth quality of direct-seeded rice. Several articles [2,8,9,10,11] discuss the possibilities of using phosphogypsum as a material to produce gypsum binders for further use for various purposes and propose a new effective technology for its utilization. The authors of [12] worked on obtaining a durable composite material based on phosphogypsum for construction use. Research [13] discusses the prospects of using phosphogypsum in road surface production. In studies [14,15,16,17], it was proposed to use phosphogypsum in chemical ring gasification processes, including synthesis gas production. In addition, developments in the field of extraction of rare earth elements from phosphogypsum are gaining widespread use [18]. The synthesis of organic–inorganic composite materials based on biochars and phosphogypsum [19,20] is accompanied with the production of effective adsorbents for antimonate ions and lead cations.

One of the possible applications of phosphogypsum is its use as a raw material for calcium sulfide synthesis [21,22,23,24,25]. Luminescent materials are produced based on CaS [26].

Several radiation phenomena that sharply deviate from the laws of thermal radiation have long been known. In some cases, substances begin to emit light at temperatures so low that the radiation cannot be explained by the transition of thermal energy into light. This includes the phenomena of the so-called “cold glow”, for example, paint covering the dials of glow-in-the-dark watches, etc. Such a glow is called luminescence. Luminescence is usually divided into the following classes: Triboluminescence is a glow arising from mechanical action, which includes numerous cases of glow from impact, friction, splitting, grinding, etc. The glow of sugar can serve as an example. Thermoluminescence is a glow caused by heating at relatively low temperatures (100–200 °C) at which there is no presence of visible thermal radiation. The glow of fluorite when heated is an example of that. Photoluminescence is a glow caused by light. There are usually two cases here: the first is when the glow continues after the end of irradiation (phosphorescence), and the second is when the glow is practically observed only during the continuation of the illumination process itself (fluorescence). For the case of illumination by X-rays, the term X-ray luminescence is used. Chemiluminescence is the result of chemical processes accompanied by light emittance without a particular increase in the reaction temperature. The glow during the oxidation of white phosphorus can serve as an example here.

Silicates, phosphates, sulfides, and other compounds are used as luminescent materials. In industry, zinc, barium, calcium, and cadmium sulfides activated with copper, silver, and rare earth elements (REE) are used. Calcium sulfide is one of the widely used luminophores [26,27].

There are direct and indirect methods for obtaining sulfide matrices. The essence of the direct synthesis method is to carry out a high-temperature reaction between two finely dispersed substances placed in a sealed ampoule filled with an inert gas. The ampoule is placed in a one- or two-zone oven. Heating is carried out gradually, but the higher the synthesis temperature, the faster the reaction proceeds and better homogenization of the final compound occurs. In general, the process of the direct synthesis of sulfides is represented by Equation (1):

xMe + yS = Me_x_S_y_ (1)

The indirect method of calcium sulfide synthesis consists of a simpler way of obtaining sulfides from salts and oxides when they react with the vapors of a sulfonating agent.

According to the studies presented in [28], alkaline earth metal sulfides were synthesized by calcining a mixture of corresponding carbonates, sulfur, and a certain amount of a reducing agent. Starch was used as a reducing agent, since, when decomposing at high temperatures, it is a source of pure carbon and does not pollute the resulting sulfide with its decomposition products.

As a rule, the synthesis of sulfide luminescent materials requires the use of analytical reagents.

The goal of this study was to obtain luminescent materials based on a calcium sulfide matrix. To obtain CaS, we used phosphogypsum.

## 2. Experiment

### 2.1. Materials

To obtain calcium sulfide, agricultural phosphogypsum with a calcium sulfate dihydrate CaSO_4_∙2H_2_O content of 99% (wt.) was used. Citric acid (С_6_Н_8_О_7_) was used as a reducing agent, in which the content of the main component was 99.5% (wt.).

### 2.2. Composite Materials Synthesis 

The synthesis was carried out according to the procedure described in detail in [29]. Phosphogypsum was pre-dried to a constant weight at a temperature of 373 K. To prepare the samples, 14 g of phosphogypsum and 4 g of a reducing agent were used, which were weighed with an accuracy of 0.01 g on an electronic balance and homogenized for 30 s. in a 0.45 kW mixer at 1500 rpm, placed in alundum crucibles in the working space of a muffle furnace, and subjected to heat treatment according to the temperature–time regimen, including heating to 140 °C for 30 min, holding for 30 min, heating to an isothermal temperature exposure at the speed of 13 K/min, exposure for 60 min, and slow cooling in the oven to room temperature. 

### 2.3. Characteristics

Various techniques were used to characterize the resulting composite materials, including X-ray diffraction (XRD), transmission electron microscopy, the Scherrer method, thermogravimetric analysis (TGA), and FT-IR spectroscopy.

The phase composition was studied using an ARL X’TRA X-ray diffractometer (monochromatized Cu-Kα radiation was used) by point-by-point scanning (0.01° step, 2 s. accumulation time at a point) in the range of 2θ from 5 °C to 90 °C. The crystallite size was calculated using the Scherrer Equation (2)

*D* = 0.94·λ/(*B*∙cosθ) (2)

where *D* is the average crystal size (nm), λ is the X-ray wavelength (nm), *B* is the peak linewidth at half height (rad), and cosθ is the cosine angle value for the peak.

To calculate the crystallite sizes, the following lines were used: line 020 for CaSO_4_·2H_2_O, CaSO_4_; line 200 for CaSO_4_·0.5H_2_O; and line 220 for CaS. The lines for calculation were chosen to avoid the phases’ reflections overlap.

The unit cell volume *V* (nm^3^) was calculated using Formulas (3) and (4):

- for monoclinic and orthorhombic phases:
  V = *a*·*b*·*c*·*sin*β, (3)
- for the cubic phase:
  *V* = *a*^3^ (4)
  where *a*, *b*, *c* are unit cell parameters (nm).

The change in sample mass *m*_s_ after heat treatment was determined using Formula (5)

*m*_s_ = (*m*_1_ − *m*_2_) · 100/*m*_1_ (5)

where *m*_1_ is the sample mass after heat treatment, calculated theoretically (g), and *m*_2_ is the practical value of the sample mass (g).

A Quanta 200 scanning microscope (FEI Company, Hilsboro, OR, USA) was used to obtain micrographs.

Elemental analysis was carried out using a Quanta 200 scanning microscope combined with an EDAX Genesis XVS 30 X-ray microanalysis system (FEI Company).

The sample’s IR spectrum was recorded on a Spectrum Two Fourier IR spectrometer (PerkinElmer, Waltham, MA, USA) equipped with a universal device for measuring total reflectance (UATR) in the frequency range of 4000–400 cm^−1^. Spectrum processing and band intensity determination were carried out using special software (Spectrum 10.6) supplied with the spectrometer.

### 2.4. Luminescent Properties Study 

**Figure 1:**
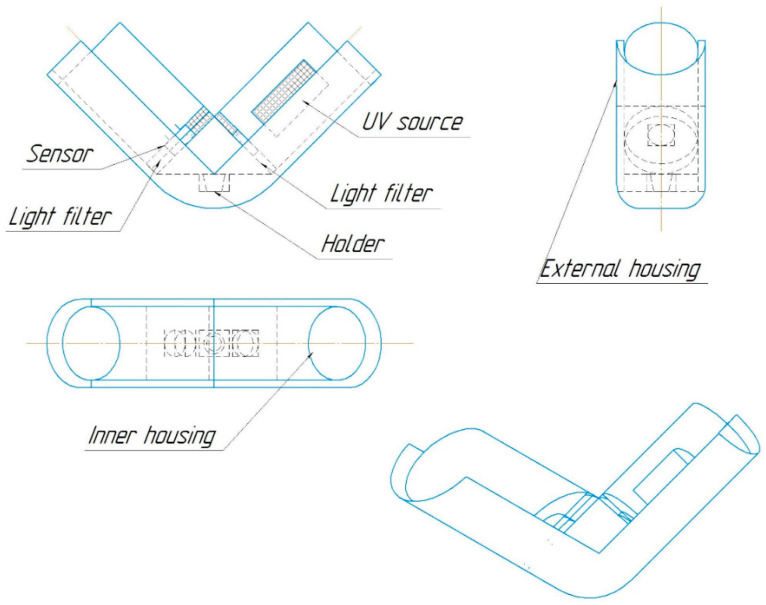
Setup scheme for measuring the light flux from the surface of a UV-irradiated sample.

For each sample, the relative luminous flux emitted by a fixed area of the sample surface was measured using the original setup [30] (Figure 1) consisting of an ultraviolet (UV) radiation source, light filters, and a recording sensor. The sample and the reference sample of a yellow YAG:Ce luminophore were placed in the installation, illuminated with radiation at a wavelength of 380 nm, then the sensor voltage drop was recorded, which occurred due to the sensor resistance change when the level of light flux illuminating its surface changed. The relative luminous flux was obtained as the ratio of the sensor voltage drop when the test sample was placed in the installation to the sensor voltage drop when the reference sample was placed in the installation.

Luminescence spectra were recorded using an FS5 spectrofluorimeter (Edinburgh Instruments, Edinburgh, UK). The absolute quantum yield was measured with an FS5 spectrofluorimeter using an SC-30 integrating sphere at λEx = 390 nm. The emission lifetime was measured using the TCSPC option of the FS5 spectrofluorimeter. The sample was excited with an EPL-375 picosecond pulsed diode laser centered at 375 nm. The instrument response function (IRF) was recorded under the described conditions by replacing the sample with a silica diffuser. Time decay data were analyzed by nonlinear curve fitting with IRF deconvolution using the Fluoracle software package F980.

## 3. Results and Discussion 

### 3.1. Samples’ Structures and Morphological Features 

**Figure 2:**
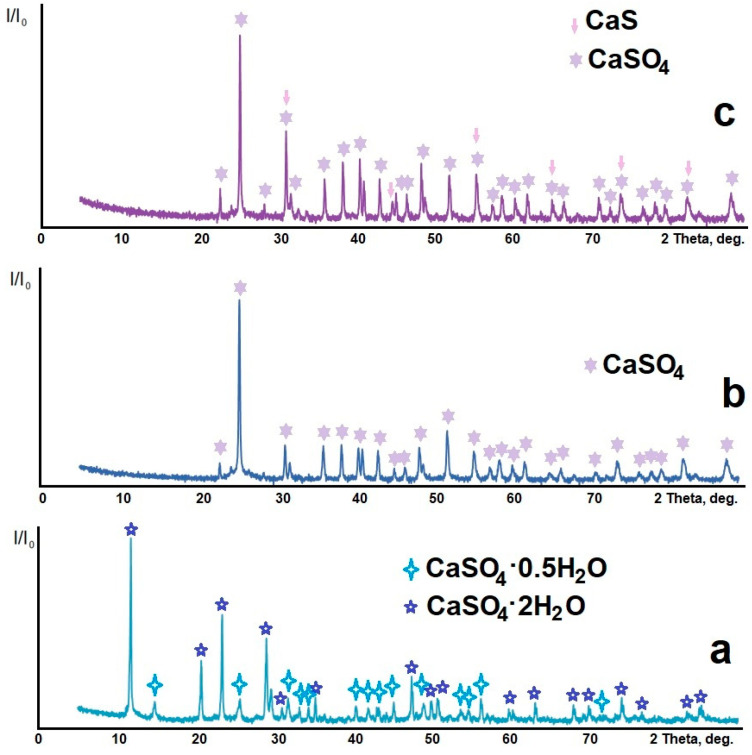
X-ray diffraction patterns of samples: (**a**) phosphogypsum dried at a temperature of 283 K; (**b**) phosphogypsum calcined at a temperature of 1073 K; (**c**) phosphogypsum calcined in the presence of a reducing agent at a temperature of 1073 K.

Figure 2 shows the X-ray diffraction patterns of the samples: sample 1—phosphogypsum dried at a temperature of 283 K to constant weight; sample 2—phosphogypsum calcined at a temperature of 1073 K for 60 min; and sample 3—phosphogypsum calcined in the presence of a reducing agent—citric acid—at a temperature of 1073 K for 60 min. Figure 2a shows an X-ray image of sample 1. The X-ray image contains reflections belonging to CaSO_4_·2H_2_O (PDF #010-70-7008) and CaSO_4_·0.5H_2_O (PDF #010-80-1235). Both compounds have a monoclinic system. The grid parameters and the crystallite sizes calculated using the Scherrer formula are presented in Table 1.

The X-ray diffraction pattern of sample 2 is shown in Figure 2b. The X-ray pattern contains reflections belonging to CaSO_4_ (PDF #010-74-2421) in the orthorhombic modification (see the grid parameters and the crystallite sizes in Table 1). The X-ray diffraction pattern of sample 3 is shown in Figure 2c. The X-ray pattern contains reflections belonging to CaSO_4_ (PDF #010-70-0909) in the orthorhombic modification and CaS (PDF #000-08-0464) in the cubic modification (see Table 1 for the grid parameters and the crystallite sizes).

As follows from the experimental data obtained, when heat treatment is carried out at a relatively low temperature, water molecules are separated in accordance with Equation (6):

CaSO_4_·2H_2_O = CaSO_4_·0.5H_2_O + 1.5H_2_O (6)

In this case, an increase in the volume of the material unit cell is observed (see Table 1). At the same time, the average crystallite size decreases significantly—by 1.9 times. When heat treated at a temperature of 1073 K, phosphogypsum completely loses crystallization water according to Equation (7)

CaSO_4_·0.5H_2_O = CaSO_4_ + 0.5H_2_O (7)

This leads to a decrease in the crystal grid volume (Table 1) and a more significant decrease (6 times) in the average size of crystallites. Heat treatment in the presence of a reducing agent leads to partial destruction of the structure with the formation of a CaSO_4_/CaS composite material according to Equations (8) and (9), and the material contains calcium sulfide clusters on the surface of calcium sulfate:

2.25CaSO_4_ + C_6_H_8_O_7_ = 2.25CaS + 6CO_2_ + 4H_2_О (8)

per carbon atom

CaSO_4_ + 2С = CaS + СО_2_ (9)

In this case, the volume of the unit cell of calcium sulfate and the average crystallite size remain virtually unchanged (Table 1). The formation of CaS crystals on the surface of CaSO_4_, which have a significantly smaller unit cell volume (Table 1), leads to an increase in the defectiveness of the composite material. Calcium sulfide crystallites are the smallest in size.

**Figure 3:**
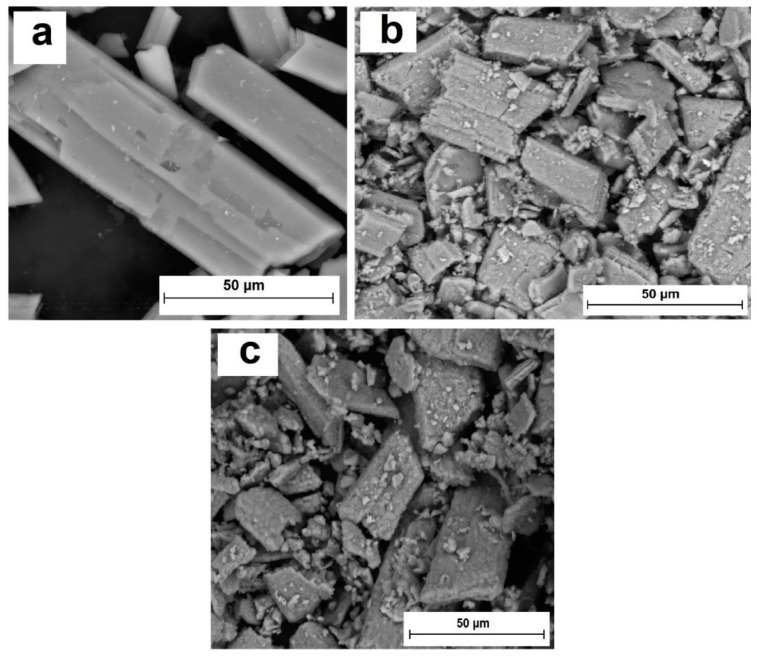
Microphotographs of samples: (**a**) phosphogypsum; (**b**) phosphogypsum calcined at a temperature of 1073 K; (**c**) phosphogypsum calcined in the presence of a reducing agent at a temperature of 1073 K.

Figure 3 shows microphotographs of the samples.

**Figure 4:**
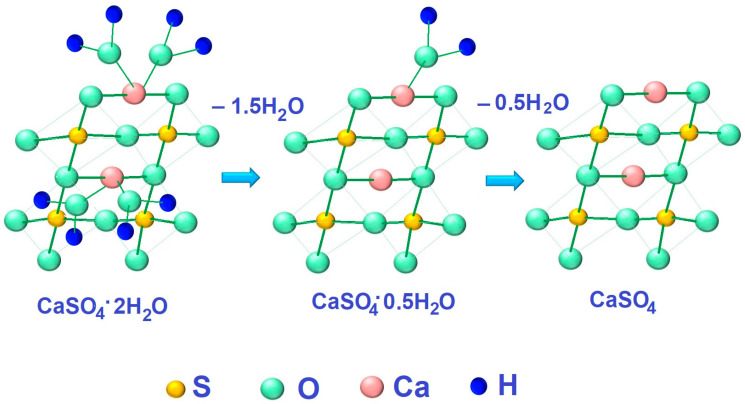
Schematic representation of the heat treatment process of phosphogypsum without a reducing agent.

Figure 3a shows a micrograph of the original phosphogypsum. The sample is represented by plate-like crystals characteristic of calcium sulfate dihydrate. After heat treatment at a temperature of 1073 K, cracks appeared on the surface of the crystals (Figure 3b). This may be due to the process of structure destruction when crystallization water is removed. The dehydration process can be represented as shown in Figure 4.

**Figure 5:**
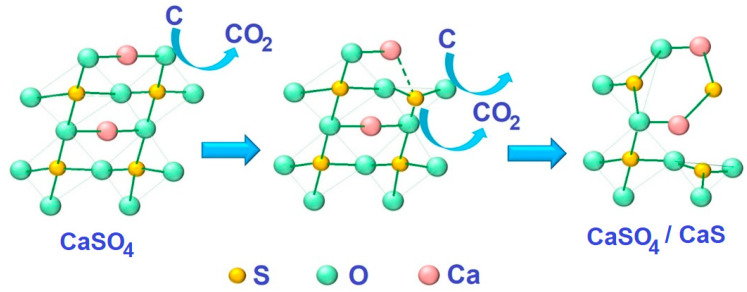
Schematic representation of the CaSO_4_/CaS composite material formation process.

During the heat treatment of phosphogypsum in the presence of a reducing agent, calcium sulfide clusters appeared on the surface of CaSO_4_ crystals (Figure 3c). Schematically, the composite material formation process can be presented as shown in Figure 5.

**Figure 6:**
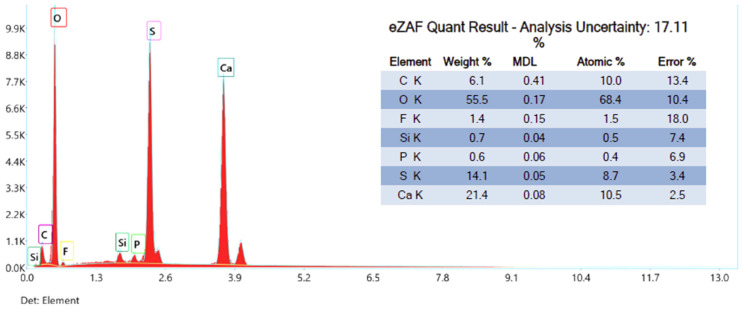
Phosphogypsum elemental analysis.

The proposed processes for the composite material formation are confirmed by the results of elemental analysis and thermogravimetric analysis. Figure 6 shows the results of phosphogypsum samples’ elemental analyses.

According to the obtained results, the main elements are oxygen (55.5% wt.), calcium (21.4% wt.), sulfur (14.1% wt.), and carbon (6.1% wt.), and impurities of fluorine, silicon, and phosphorus are also present.

**Figure 7:**
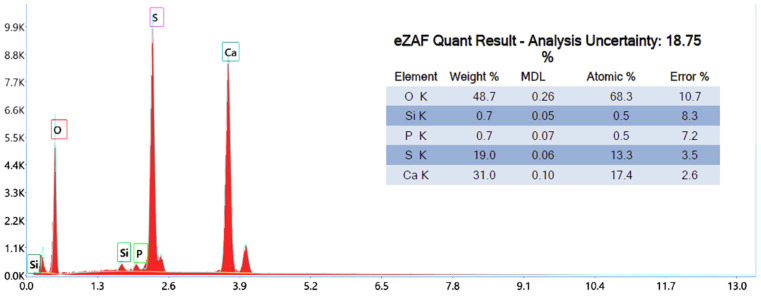
Elemental analysis of the obtained material.

Figure 7 shows the results of an elemental analysis of a phosphogypsum sample subjected to heat treatment with a reducing agent. According to the results obtained, the elemental content is as follows: oxygen (48.7% wt.), calcium (31.0% wt.), and sulfur (19.0% wt.).

Compared to the original phosphogypsum sample that was not heat treated with a reducing agent (Figure 6), a decrease in the proportion of oxygen and an increase in the proportion of calcium and sulfur were noted, which means that calcium sulfate was partially reduced to sulfide during the heat treatment.

**Figure 8:**
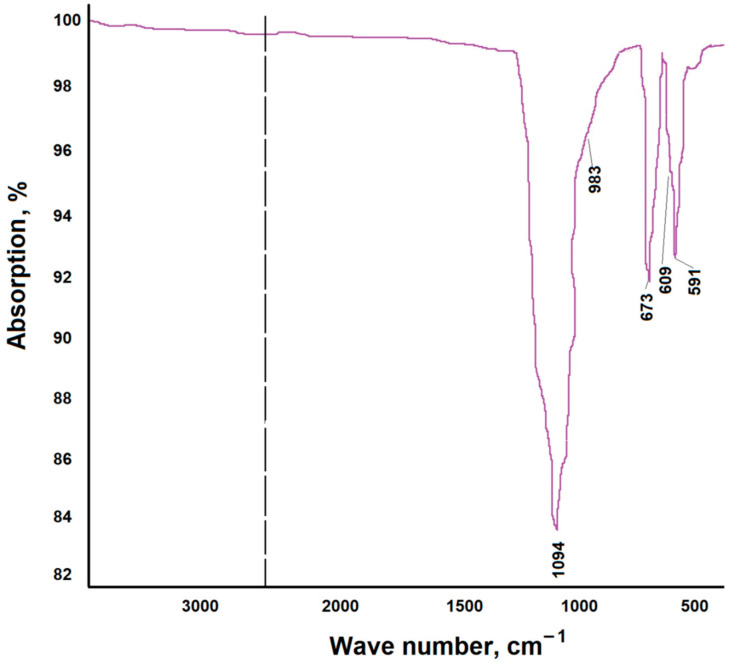
IR spectrum of the reduced phosphogypsum sample.

The IR spectrum of the reduced phosphogypsum sample (sample 3) is shown in Figure 8. The IR spectrum consists of strong absorption bands in the regions of 1100–1000 cm^−1^ and 700–550 cm^−1^, which belong to the ν3 and ν4 vibrations of the SO_4_^2−^ ion [31].

**Figure 9:**
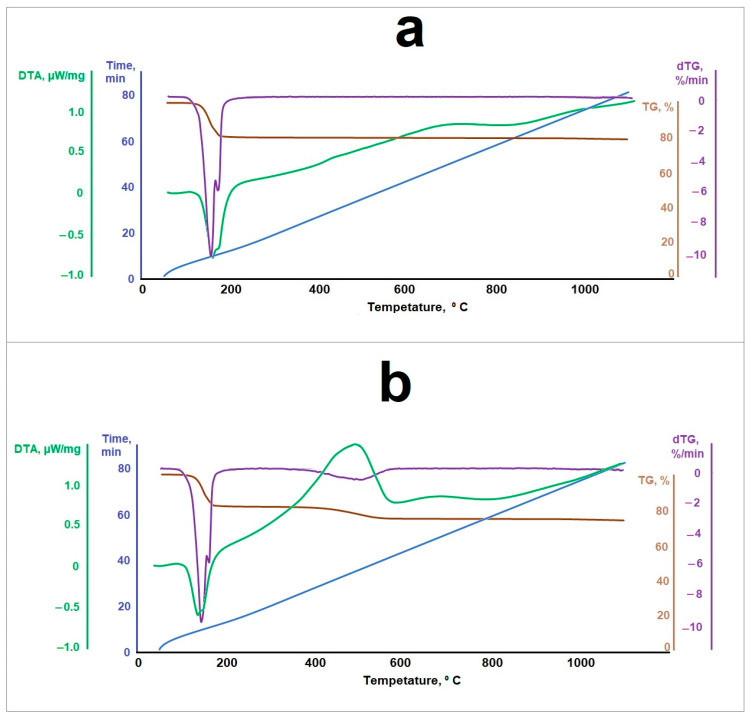
Samples’ thermogravimetric analysis data for (**a**) phosphogypsum and (**b**) phosphogypsum mixed with a reducing agent.

**Figure 10:**
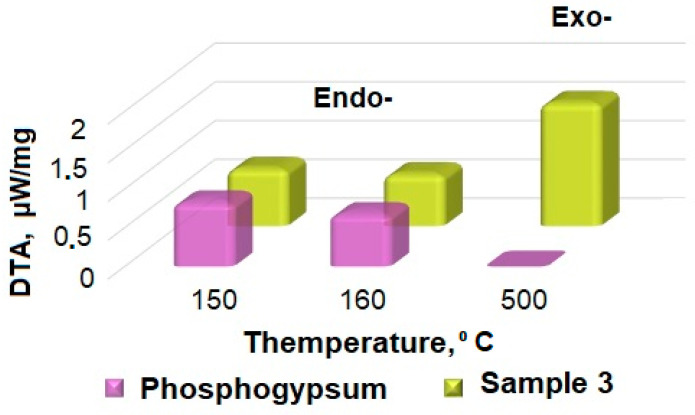
Thermogram analysis results.

The thermogravimetric analysis results for the samples of the original phosphogypsum and the phosphogypsum mixed with a reducing agent in the optimal molar ratio are shown in Figure 9. Analysis of the thermograms is shown in Figure 10 and Table 2.

As follows from the obtained data, a phosphogypsum sample, when heated, had two endothermic effects—at the temperatures of about 424 K and about 440 K—and the sample lost about 20% in mass. Further, the value of the sample mass did not change. These effects can be associated with the separation of crystallization water in accordance with Equations (6) and (7). When phosphogypsum is heated in the presence of a reducing agent, in addition to the indicated two endothermic effects at temperatures of 421 K and 429 K, respectively, a broad exothermic effect is observed at a temperature of 773 K. It may be associated with the thermal decomposition of the reducing agent. The dependence of the measurement of sample 3’s mass on temperature has two inflection points associated with the crystallization water loss (the interval and magnitude of the inflection is similar to the pure phosphogypsum sample) and the reducing agent decomposition processes according to Equations (6) and (7) in the temperature range of 650–770 K.

### 3.2. Characteristics of the Materials’ Luminescences

**Figure 11:**
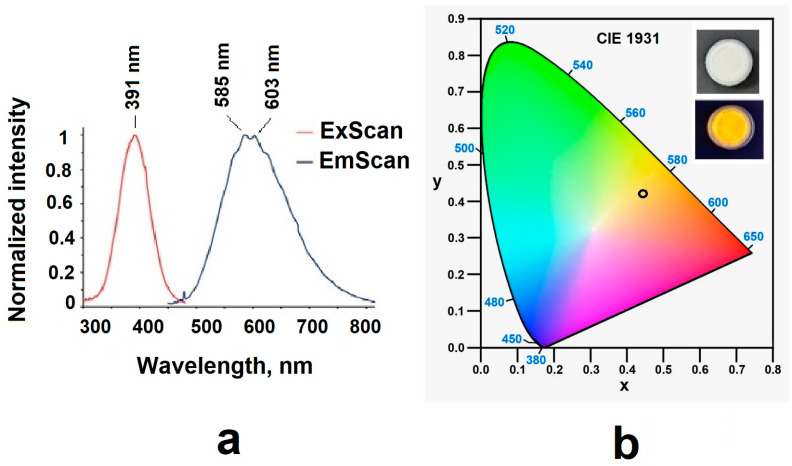
(**a**) Excitation and emission spectra and (**b**) color diagram for reduced samples.

**Figure 12:**
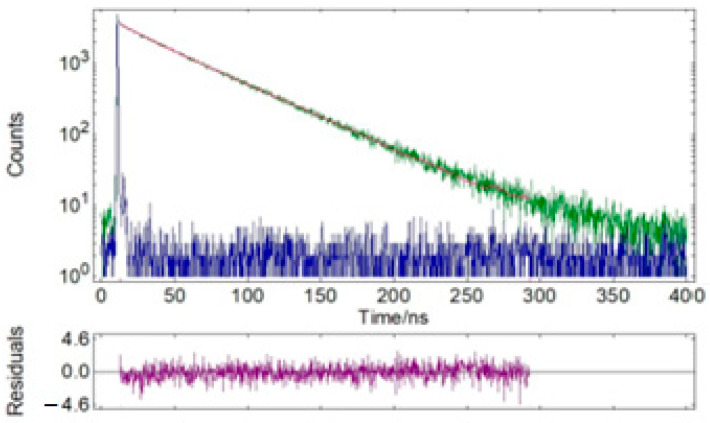
Time-resolved fluorescence lifetime decay profiles of solid powder sample 3 (green) and instrumental response function (IRF, blue). λ_ex_ = 375 nm, λ_em_ = 585 nm.

**Figure 13:**
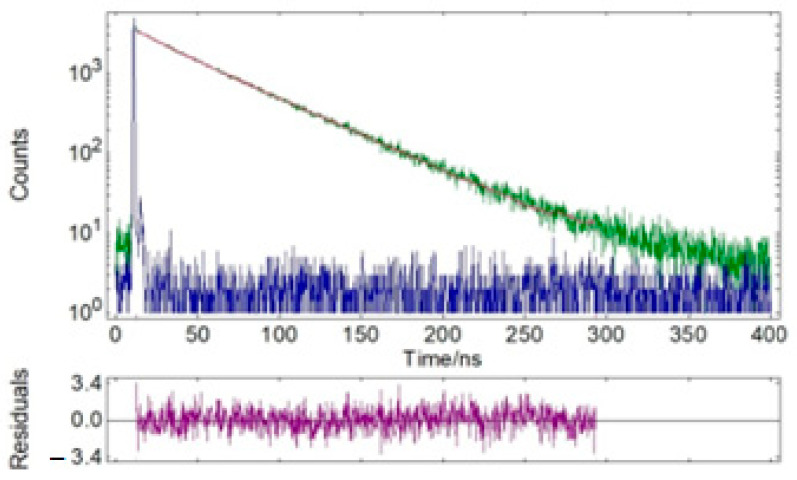
Time-resolved fluorescence lifetime decay profiles of solid powder sample 3 (green) and instrumental response function (IRF, blue). λ_ex_ = 375 nm, λ_em_ = 603 nm.

The emission and excitation spectra of sample 3 were recorded; the data are presented in Table 3 and Figure 11. The sample had orange emission in the range of 500–750 nm with a quantum yield of 0.17. Experimental results showed that the sample’s decomposition process in the solid state consisted of two components with a predominant contribution of the long-lived component (~46 ns) (Table 3 and Table 4; Figure 12 and Figure 13).

### 3.3. Selection of the Optimal Heat Treatment Duration for the Luminescent Material Synthesis

**Figure 14:**
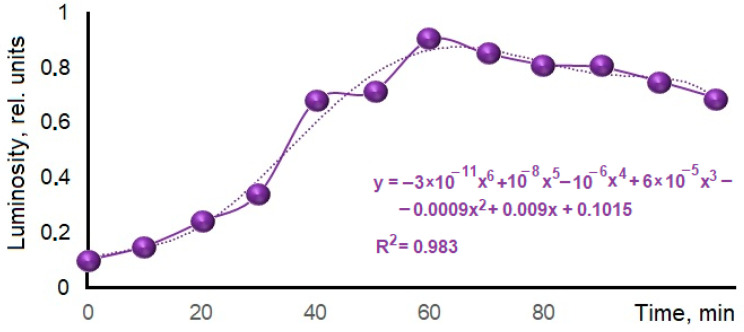
Dependence of samples’ relative luminosities on the treatment duration.

To determine the optimal heat treatment time, samples of phosphogypsum weighing 17.2 g and a reducing agent were subjected to heat treatment according to the following regimen: samples were heated at a rate of 13 K/min to a temperature of 1073 K; upon reaching the pre-assigned temperature, some individual samples were moved into a cooling chamber made of thermal insulating material every 10 min, where they slowly cooled to room temperature. After this, the samples were reweighed, crushed in a mortar, and the relative luminous flux emitted by the fixed area of sample surfaces was measured. The results are shown in Figure 14.

The obtained data indicate that at the given temperature, the holding time of 60 min was optimal for obtaining luminescent material. It can be assumed that a shorter holding time is not enough for the reduction process to occur, which is also evidenced by the insufficient mass loss compared to the calculated one and the traces of unreacted coal observed in the calcined samples. A longer calcination time leads to a reverse oxidation reaction. This is evidenced in the samples’ mass increases after calcination at heat treatment temperatures of 1173 K and 1273 K (Table 5). The presence of the first maximum in the calcined sample’s mass increase at the heat treatment temperature of 1173 K may be associated with a denser calcium sulfate modification formation under experimental conditions.

### 3.4. Selection of the Optimal Heat Treatment Temperature for the Luminescent Material Synthesis

To determine the optimal heat treatment temperature, phosphogypsum weighing 17.2 g and the reducing agent were subjected to heat treatment according to the following modes: The samples were heated at a rate of 13 K/min to the calcination temperature, which was 1073 K, 1173 K, or 1273 K for the three samples, respectively. A holding period of 60 min followed when the calcination temperature was reached. At the end of the heat treatment, the samples were cooled together with the furnace to room temperature. The results of the samples’ relative luminous flux measurements are presented in Table 6.

According to the obtained results, the best luminescent quality was exhibited by phosphogypsum samples which were heat-treated in the presence of citric acid at 1073 K; the maximum value of the relative luminous flux was achieved at the reducing agent mole fraction of 37%. Under experimental conditions, at any heat treatment temperature, a decrease in the sample’s luminescence was observed with the introduction of a significant amount of the reducing agent. This effect may be associated with the formation of carbon-containing compounds that were not detectable by X-ray diffraction but which affect the samples’ luminosity. For the heat treatment temperature of 1173 K, the maximum luminosity of the samples was achieved at a reducing agent mole fraction of 49%. For the temperature of 1273 K, maximum luminosity was achieved at a mole fraction of 74%. This may be due to the formation of a more stable modification of CaSO_4_ at higher heat treatment temperatures, the reduction of which requires more organic matter.

### 3.5. Study of the Simultaneous Influence of Temperature and Calcination Time on the Luminescent Quality of the Synthesized Material

To study the simultaneous influence of temperature and calcination time on the luminescent quality of the material synthesized, samples’ heat treatments were carried out with a simultaneous increase in temperature and a decrease in the calcination time, as well as with a simultaneous decrease in temperature and an increase in the calcination time in comparison to the optimal values adopted for the samples. The calcination temperatures and times are given in Table 7.

The obtained data indicate that an increase in temperature with a simultaneous decrease in heat treatment time, as well as a decrease in temperature with a simultaneous increase in heat treatment time, led to a decrease in the luminescent properties of the synthesized material compared to the optimal values, which are a temperature of 1073 K and a duration of 60 min.

## 4. Conclusions

The process of CaSO_4_/CaS composite material formation during the reduction of phosphogypsum with citric acid has been studied. The composite structure formation mechanism is proposed.

It is shown that during the process, the oxygen proportion decreases and the calcium and sulfur proportions in the sample increase, which indicate the partial reduction of calcium sulfate to sulfide.

The reduced sample was found to emit orange radiation in the range of 500–750 nm with a quantum yield of 0.17. Experimental results showed that the sample decomposition process in the solid state consisted of two components with a predominant contribution from the long-lived component (~46 ns).

The optimal conditions for producing luminescent materials by reducing phosphogypsum with citric acid were determined to be heat treatment at a temperature of 1073 K, a holding time of 60 min, and a reducing agent mole fraction of 37%.

It was found that an increase in temperature with a simultaneous decrease in heat treatment time, as well as a decrease in temperature with a simultaneous increase in heat treatment time, led to a decrease in the luminescent properties of the synthesized material compared to optimal values.

The results can be used to develop technology for recycling large-tonnage waste from the chemical industry into luminescent materials.

## Figures and Tables

**Table 1:** Crystallographic parameters of samples.

Sample	Phase	Grid Parameters, nm			β	Volume, nm^3^	*D*, nm
		*a*	*b*	*c*		*V*	
Sample 1	CaSO_4_·2H_2_O	5.67	15.11	6.49	118.5	489	535
	CaSO_4_·0.5H_2_O	12.02	6.93	12.67	90.2	1055	285
Sample 2	CaSO_4_	6.23	6.98	6.97	–	303	47
Sample 3	CaSO_4_	6.99	7.00	6.24	–	305	49
	CaS	5.69	–	–	–	184	43

**Table 2:** Thermogravimetric analysis results.

Sample	Character	Phosphogypsum		Sample 3	
		Temperature, K	Value, µW/mg	Temperature, K	Value, µW/mg
Peak 1	Endo	424	0.84	421	0.74
Peak 2	Endo	440	0.69	429	0.67
Peak 3	Exo	-	-	773	1.59

**Table 3:** Photoluminescent data for compound sample in powder at r.t.

Excitation, λ_em_ [nm]	Emission, λ_em_ [nm]	τ_avr,_ [ns]/χ^2^	Φ_F_
391	585	46.02/1.110	0.17
	603	45.81/1.034	

**Table 4:** Detailed data of the fluorescence lifetime measurements of sample 3: τ—lifetime, *f*—fractional contribution, τ_avg_—average lifetime, χ^2^—chi-squared distribution.

Solid						
λ_em_ [nm]	τ_1,_ [ns]	*f*_1_, %	τ_2_ [ns]	*f*_2_, %	τ_avg,_ [ns]	χ^2^
585	16.22	5.3	47.70	94.7	46.02	1.034
603	15.06	5.2	47.49	94.8	45.81	1.110

**Table 5:** Changes in the samples’ masses depending on the introduced reducing agent amount and heat treatment temperature.

Reducing Agent Fraction, %	Heat Treatment Temperature, К		
	1073	1173	1273
6.3	0.00	0.07	0.07
12.6	0.00	0.64	0.07
18.9	0.14	1.00	0.14
25.2	0.29	0.07	0.29
37.8	0.50	0.21	0.50
50.4	0.86	0.43	0.93
63.0	1.21	0.71	3.14
75.6	1.29	0.14	2.29

**Table 6:** Results of phosphogypsum heat treatment with different reducing agent contents at different temperatures.

Reducing Agent Mass, g	Reducing Agent Mole Fraction, %	Relative Luminous Flux (Rel. Units) at Treatment Temperature		
		1073 К	1173 К	1273 К
1.10	6.40	0.24	0.10	0.10
2.10	12.21	0.39	0.12	0.10
4.30	25.00	0.86	0.35	0.15
6.40	37.21	0.93	0.75	0.15
8.50	49.42	0.91	0.90	0.15
10.70	62.21	0.90	0.88	0.10
12.80	74.42	0.84	0.80	0.26
17.10	99.42	0.63	0.60	0.21

**Table 7:** Conditions for phosphogypsum samples’ heat treatments with citric acid.

Reducing Agent Mass, g	Reducing Agent Mole Fraction, %	Relative Luminous Flux (Rel. Units)		
		973 К, 90 min	1073 К, 60 min	1173 К, 30 min
4.30	50.59	0.38	0.86	0.10
6.40	75.29	0.41	0.93	0.15
8.50	100.00	0.51	0.91	0.72
10.70	125.88	0.46	0.90	0.90
12.80	150.59	0.44	0.84	0.87
17.10	201.18	0.41	0.63	0.74

## Data Availability

Data are contained within the article.
